# Sonographic Findings of Left Ventricular Dysfunction to Predict Shock Type in Undifferentiated Hypotensive Patients: An Analysis From the Sonography in Hypotension and Cardiac Arrest in the Emergency Department (SHoC-ED) Study

**DOI:** 10.7759/cureus.16360

**Published:** 2021-07-13

**Authors:** Sam Keefer, Paul Atkinson, Kavish Chandra, Ryan J Henneberry, Paul A Olszynski, Mandy Peach, Laura Diegelmann, Hein Lamprecht, Melanie Stander, David Lussier, Chau Pham, James Milne, Jacqueline Fraser, David Lewis

**Affiliations:** 1 Faculty of Medicine, Dalhousie University, Halifax, CAN; 2 Emergency Medicine, Horizon Health Network, Saint John, CAN; 3 Emergency Medicine, Dalhousie Medicine New Brunswick, Saint John, CAN; 4 Emergency Medicine, Dalhousie University, Halifax, CAN; 5 Emergency Medicine, University of Saskatchewan, Saskatoon, CAN; 6 Emergency Medicine, Dalhousie University, Saint John, CAN; 7 Emergency Medicine, University of Maryland, Baltimore, USA; 8 Emergency Medicine, Stellenbosch University, Cape Town, ZAF; 9 Emergency Medicine, Mediclinic, Cape Town, ZAF; 10 Emergency Medicine, University of Manitoba, Winnipeg, CAN; 11 Emergency Medicine, University of Manitoba, Winnipeg, CAN; 12 Family Medicine, Fraser Valley Health, Vancouver, CAN; 13 Emergency Medicine, Saint John Regional Hospital, Saint John, CAN

**Keywords:** emergency medicine, point of care ultrasound, shock, hypotension, left ventricular function

## Abstract

Introduction

Patients that present to the emergency department (ED) with undifferentiated hypotension have a high mortality rate. Hypotension can be divided into four categories: obstructive, hypovolemic, distributive, and cardiogenic. While it is possible to have overlapping or concomitant shock states, being able to differentiate between cardiogenic shock and the other categories is important as it entails a different treatment regime and extra cautions. In this secondary analysis, we investigate if using focused cardiac ultrasonography (FOCUS) to determine left ventricular dysfunction (LVD) can serve as a reliable test for cardiogenic shock.

Methods

We prospectively collected FOCUS findings performed in 135 ED patients with undifferentiated hypotension as part of an international study. Patients with clearly identified etiologies for hypotension were excluded, along with other specific presumptive diagnoses. LVD was defined as the identification of a generally hypodynamic left ventricle in the setting of shock. FOCUS findings were collected using a standardized protocol and data collection form. All scans were performed by emergency physicians trained in ultrasound. Final shock type was defined as cardiogenic or noncardiogenic by independent specialist blinded chart review.

Results

In our findings, 135 patients had complete records for assessment of left ventricular function and additional follow-up data and so were included in this secondary analysis. The median age was 56 years and 53% of patients were male. Disease prevalence for cardiogenic shock was 12% and the mortality rate was 24%. The presence of LVD on FOCUS had a sensitivity of 62.50% (95% confidence interval 35.43% to 84.80%), specificity of 94.12% (88.26% to 97.60%), positive likelihood ratio (LR) 10.62 (4.71 to 23.95), negative LR 0.40 (0.21 to 0.75) and accuracy of 90.37% (84.10% to 94.77%) for detecting cardiogenic shock.

Conclusion

Detecting left ventricular dysfunction on FOCUS may be useful in the early identification of cardiogenic shock in otherwise undifferentiated hypotensive adult patients in the emergency department.

## Introduction

Patients who present to the emergency department (ED) with undifferentiated hypotension have high rates of morbidity and mortality [1]. The four major categories into which undifferentiated shock can be divided are hypovolemic, cardiogenic, distributive, and obstructive [2]. While at times concomitant shock states are seen, they are generally approached with different treatment regimes. [2]. Cardiogenic shock is the result of severely impaired myocardial performance which leads to hypoxia, diminished cardiac function, and end-organ hypoperfusion [3].

Differentiating between cardiogenic and noncardiogenic shock in patients with undifferentiated shock can help physicians deliver appropriate treatment [4]. This is important as treatment choices such as fluid resuscitation versus vasopressors are recommended for the different categories. The high mortality seen in cardiogenic shock also highlights the importance of having a rapid and accurate diagnosis to guide treatment [5].

There are a variety of methods for differentiating between the above types of shock [6]. One commonly used technique is point-of-care ultrasonography (PoCUS). This has been demonstrated to be an effective adjunct to the bedside evaluation of a patient that can help differentiate between types of shock [7]. Shock PoCUS protocols, such as the rapid ultrasound for shock and hypotension (RUSH) exam, have been used in the determination of shock type [4]. These protocols have been shown to have particular utility as a rule-in test for the various causes of shock [4]. Previously the use of focused ultrasound as a diagnostic tool in emergency medicine has focused on the detection of pericardial fluid and global cardiac activity. The use of focused cardiac ultrasound in emergency medicine has progressed to include additional views and a more structured approach to the assessment of the left ventricle [8]. Sonographic assessment of left ventricular function has been proposed as a method of determining the etiology of shock [9]. In this secondary analysis, we will examine whether focused cardiac ultrasound (FOCUS) assessment of left ventricle function can serve as a reliable predictor for cardiogenic shock in adult emergency department patients with undifferentiated hypotension.

## Materials and methods

This is a secondary analysis of data collected during an international randomized controlled trial that was conducted in six centers across North America and South Africa. The original study included 273 patients [1]. These 273 participants were randomized to a control group (n=135) and an experimental group receiving a structured PoCUS protocol (n=138). We prospectively collected the PoCUS findings for 135 ED patients with undifferentiated hypotension in the experimental group that received PoCUS. The inclusion criteria used selected adult patients (aged 19 years or older) identified as having a sustained systolic blood pressure (SBP) less than 100 mmHg or a shock index greater than 1.0. Patients with clearly identified etiologies for hypotension were excluded from the study, along with other specific presumptive diagnoses including patients with ectopic pregnancy or aortic aneurysm, evidence of differentiated hypotension as indicated by cardiopulmonary resuscitation (CPR) or other advanced cardiac life support interventions; a history of significant recent trauma; acute myocardial infarction (AMI); another clear mechanism or etiology for the hypotension or shock such as gastrointestinal bleeding. 

Focused cardiac ultrasonography (FOCUS) was used to determine cardiac function. Left ventricular dysfunction (LVD) was defined as the identification of a generally hypodynamic left ventricle in the setting of undifferentiated shock. FOCUS findings were collected using a standardized PoCUS protocol and data collection form. All scans were performed by PoCUS-trained emergency physicians. The final shock type was defined as cardiogenic or non-cardiogenic by two independent specialists conducting blinded chart reviews. Comparative statistics were then used to compare the results of the FOCUS assessment for LVD with the final shock type. The study was registered at ClinicalTrials.gov (registration number NCT01419106) and all sites received local research ethics board (REB) approval. The study was completed in line with the Standards for Reporting Diagnostic Accuracy (STARD) checklist [10]. The work has been previously presented at the Canadian Association of Emergency Physicians' national scientific conference, 2020, and the abstract was published in the proceedings of that meeting [11].

## Results

Of the 138 patients included in the ultrasound arm of the SHoC-ED study [1], 135 patients had complete records for assessment of left ventricular function and additional follow-up data and were included in this secondary analysis. The median age of patients was 56 years of age and 53% of the patients were male. The observed disease prevalence in this group was 12% and the mortality rate was found to be 24% (see Table 1). 

**Table 1:** Baseline demographic profile of study participants and primary outcome PoCUS: point-of-care ultrasound; CI: confidence intervals; n: number; IQR: inter-quartile range; ED: emergency department; HR: heart rate; SBP: systolic blood pressure

Group Characteristics	
Total participants receiving PoCUS (n)	138
Total with complete data and follow up (n)	135
North America n (%; 95% CI)	90 (65.2%; 56.6 to 73.1%)
South Africa n (%; 95% CI)	48 (34.8%; 26.8 to 43.3%)
Male n (%; 95% CI)	73 (52.9%; 44.2 to 61.4%)
Age in years: median (IQR)	56 (53.4 to 59.8)
SBP in mmHg: Median (IQR)	91.0 (88.5 to 94.2)
HR: median (IQR)	106.5 (102.4 to 111.8)
Respiratory rate: median (IQR)	24.3 (22.3 to 26.0)
Temp in deg celcius: median (IQR)	36.7 (36.5 to 36.9)
Final diagnosis cardiogenic shock n (%; 95% CI)	16 (11.85%; 6.93 to 18.53%)
Final diagnosis non-cardiogenic shock n (%; 95% CI)	119 (88.15%; 81.52 to 92.67%)

Additionally, 17 patients had a finding of left ventricular dysfunction (hypodynamic LV) on FOCUS with 118 having either normal or hyperdynamic function recorded. For the detection of cardiogenic shock, the presence of LVD on FOCUS had a sensitivity of 62.50% (95% CI 35.43% to 84.80%), and a specificity of 94.12% (88.26% to 97.60%). FOCUS had a positive likelihood ratio (LR) of 10.62 (4.71 to 23.95), and a negative-LR of 0.40 (0.21 to 0.75). The accuracy was found to be 90.37% (84.10% to 94.77%) for identifying cardiogenic shock (Table 2).

**Table 2:** Diagnostic test performance of focused cardiac ultrasound detection of left ventricular dysfunction as a determinant of cardiogenic shock in undifferentiated hypotensive adults.

Statistic	Value	95% Confidence Interval
Sensitivity	62.50%	35.43% to 84.80%
Specificity	94.12%	88.26% to 97.60%
Positive likelihood ratio	10.62	4.71 to 23.95
Negative likelihood ratio	0.4	0.21 to 0.75
Disease prevalence	11.85%	6.93% to 18.53%
Positive predictive value	58.82%	38.79% to 76.30%
Negative predictive value	94.92%	90.83% to 97.24%
Accuracy	90.37%	84.10% to 94.77%

## Discussion

The results of this secondary analysis indicate that focused bedside sonographic assessment of the left ventricle looking for left ventricular dysfunction represents a useful tool in determining if undifferentiated hypotensive shock is cardiogenic in nature. This is indicated by the high specificity of 94% and a positive predictive value of 10.62, meaning that FOCUS is an effective rule-in test for cardiogenic shock. Overall, FOCUS to determine left ventricular function has moderate predictive value for cardiogenic shock, with potential clinical utility in undifferentiated hypotension. However, a sensitivity of 62.5% indicates that this method is likely not as reliable when used to rule out this specific category of shock. The relatively high accuracy of 90.37% indicates that the findings of FOCUS were consistent with the final blinded specialist chart reviews. 

This rapid focused bedside assessment represents a potentially useful tool to initially determine the correct treatment path for undifferentiated hypotensive adults, by providing physicians with an early likely category of shock. While further testing utilizing comprehensive echocardiography can be used later on in the treatment pathway, this method represents a simple, rapid, and straightforward tool that can help physicians tailor their initial treatment. The observed mortality rate of 24% in this cohort demonstrates the importance of having a rapid and accurate way of identifying shock type such that appropriate treatment regimes can be given, with the aim of decreasing mortality. Rather than always completing a multi-organ structured shock PoCUS protocol, perhaps a focused approach, using ultrasound in a Bayesian manner to address specific questions (such as cardiogenic vs non-cardiogenic shock) is acceptable. Building a set of clinical questions relevant to the clinical situation, with a hierarchy of questions based on known disease prevalence, risk factors and the potential utility of ultrasound to differentiate or diagnose in that setting would help to individualize sonography in hypotensive patients. This approach is further outlined in the Sonography in Hypotension and Cardiac Arrest (SHoC) protocol [12].

Limitations

This study is limited in that it is a secondary analysis with innate disadvantages such as data not being collected with the expressed purpose of answering this question. However, the use of a standardized data collection form validates the approach. This was a relatively small analysis with a limited number of participants that were found to be in cardiogenic shock. The exclusion criteria removed patients with several shock etiologies such as acute myocardial infarction, suspected aortic aneurysm, and other pathologies, indicating potential selection bias. As these pathologies do not represent true undifferentiated shock, their exclusion is reasonable.

## Conclusions

Focused cardiac ultrasound identification of left ventricular dysfunction has a moderate predictive value for determining the presence of cardiogenic shock in adult emergency department patients with undifferentiated hypotension. With the significant challenges and risks associated with the management of cardiogenic shock, the high performance of FOCUS as a rule-in test suggests that early identification of left ventricular dysfunction may be useful for earlier consultation and tailored management appropriate for the individual patient and scenario.
